# Establishing Standardized Uptake Value Threshold for Urologic Malignancies With New Analytic Software Based on Extended Single-Photon Emission Computed Tomography-Processed Bone Scintigraphy Images

**DOI:** 10.7759/cureus.95109

**Published:** 2025-10-21

**Authors:** Shunsuke Watanabe, Daisuke Motoyama, Keita Tamura, Satoshi Goshima, Teruo Inamoto

**Affiliations:** 1 Department of Urology, Hamamatsu University School of Medicine, Hamamatsu, JPN; 2 Department of Developed Studies for Advanced Robotic Surgery, Hamamatsu University School of Medicine, Hamamatsu, JPN; 3 Department of Radiology, Hamamatsu University School of Medicine, Hamamatsu, JPN

**Keywords:** bone metastasis, gi-bone, suv, urologic oncology, xspect

## Abstract

Accurate reference thresholds are critical for reliable interpretation and quantification in nuclear medicine imaging. In recent years, single-photon emission computed tomography (SPECT)/CT systems capable of standardized uptake value (SUV) analysis have become commercially available; however, most SPECT/CT systems lack the capability to perform SUV analysis. The GI-BONE system (AZE Co., Ltd., Tokyo, Japan) is a new vendor-free software that incorporates a tool for calculating the becquerel calibration factor (BCF), which converts SPECT images from a count-based scale to a radioactivity concentration scale comparable to that of PET. This study aimed to first describe a way for defining optimal SUV threshold specific to GI-BONE analysis using extended SPECT (xSPECT), which is characterized by its capability to facilitate more precise diagnosis and evaluation through enhanced analytical refinement to conventional SPECT imaging. This approach can guide clinicians in formulating their own disease system-specific thresholds, an indispensable step in adapting GI-BONE as a newly applicable and reliable mode of xSPECT verification technology.

## Introduction

Bone metastases from urologic malignancies pose significant diagnostic challenges, often requiring advanced imaging for accurate detection and management. One of the most widely used quantitative metrics is the standardized uptake value (SUV). It reflects the degree of radiotracer accumulation normalized by injected dose and body weight, providing a quantitative index of metabolic activity that enables reproducible comparison across patients and scanners. However, its application requires careful calibration and threshold setting to distinguish between normal and pathological uptakes. Recent studies showed that active bone metastases exhibit significantly higher SUVs than degenerative changes [1], supporting the use of SUV metrics for lesion discrimination. The uptake intensity in bone single-photon emission computed tomography (SPECT) images, when measured as counts using a volume of interest (VOI), is dependent on factors such as administered dose and body weight, making accurate comparative evaluation challenging. However, SUV-normalized SPECT images provide a quantitative metric of uptake intensity that accounts for these factors, enabling longitudinal comparisons of uptake intensity within the same subject and facilitating inter-patient comparisons. With the introduction of extended SPECT (xSPECT) technology, which enhances spatial resolution and contrast by integrating CT-based attenuation correction and iterative reconstruction algorithms, SUV values obtained may differ significantly from those using conventional methods. Consequently, older SUV thresholds may not apply to xSPECT-processed images. Urologic malignancies, such as prostate, kidney, and bladder cancer, frequently metastasize to bone, making the ability to accurately establish SUV thresholds critical for effective disease management.

This technical report describes the way to establish an SUV threshold for GI-BONE (AZE Co., Ltd., Tokyo, Japan) analysis, a specialized quantitative approach for assessing bone involvement in urologic malignancies, based on xSPECT bone scintigraphy.

## Technical report

Patient selection

We retrospectively analyzed bone scintigraphy images from patients with prostate, bladder, or renal cancer acquired between January and March 2025. The study was approved by the institutional review board (IRB number: 21-088).

Imaging protocol and xSPECT processing

**Figure 1:**
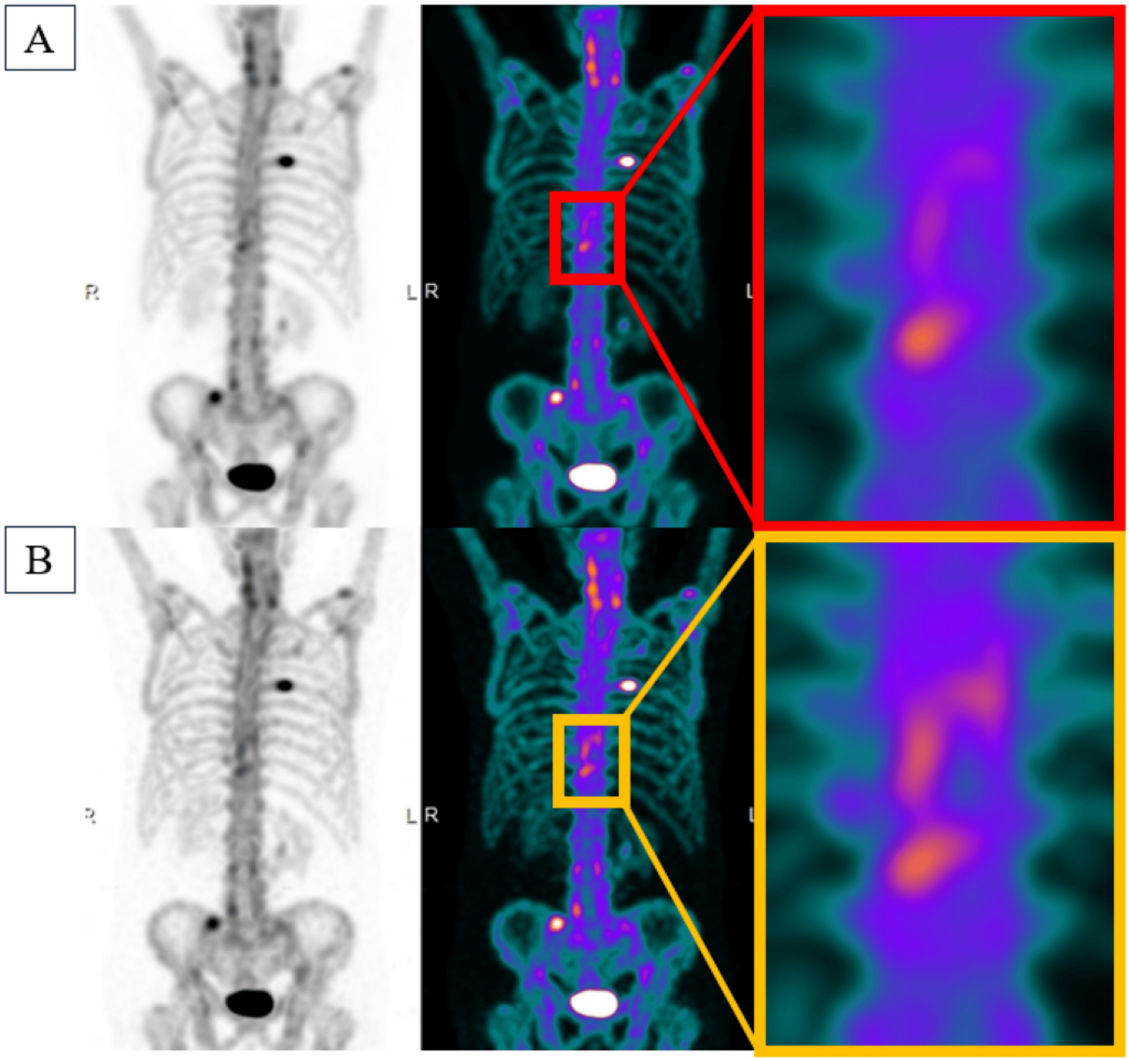
Bone scintigraphy images These are the images processed using the conventional method (A) and the images processed using xSPECT (B). The xSPECT-processed images exhibit higher contrast than those processed using the conventional method. xSPECT, extended single-photon emission computed tomography (SPECT)

All patients received 740 MBq of 99mTc-hydroxymethylene diphosphonate (99mTc-HMDP) intravenously. Whole-body planar and SPECT/CT imaging was performed three hours post-injection using a Symbia Intevo (catalog number S-747, SIEMENS Healthineers Co., Ltd., Tokyo, Japan), which incorporates xSPECT reconstruction. xSPECT Broad Quantification is an advanced reconstruction and quantification algorithm specifically designed to enable standardized, accurate activity measurement across a wide range of isotopes and collimators. Unlike conventional SPECT reconstruction methods, which focus primarily on relative image contrast, xSPECT Broad Quantification integrates a system-specific calibration factor and advanced modeling of detector and collimator physics into the iterative reconstruction process. This approach allows direct conversion of voxel values into activity concentrations expressed in kBq/mL, thereby enabling harmonized and reproducible quantification independent of the acquisition conditions. As a result, the images obtained exhibit higher contrast compared to conventional imaging methods (Figure 1).

GI-BONE bone scintigraphy quantification software

The GI-BONE (Nihon Medi-Physics Co., Ltd., Tokyo, Japan) is post-processing software designed for the quantitative assessment of bone scintigraphy. It incorporates a tool for calculating the BCF, which enables the conversion of SPECT images from a count-based scale to a radioactivity concentration scale equivalent to that used in PET imaging. The BCF is determined by scanning a cylindrical phantom with a known radioactivity concentration. This BCF is subsequently applied to clinical data, allowing clinical SPECT images to be converted from a count-based scale to images reflecting radioactivity concentrations comparable to those in PET imaging. The resulting SPECT images, expressed in terms of radioactivity concentration, can be normalized to SUV based primarily on administered dose and patient body weight, thereby enabling quantitative assessment of uptake intensity [2]. Traditionally, bone scintigraphy has relied on qualitative interpretation, which limits its ability to objectively monitor changes over time or assess the treatment response. GI-BONE addresses these limitations by providing quantitative data, achieving high reproducibility and reliability. GI-BONE automatically places the VOI on SUV values exceeding the set threshold and measures SUV max, SUV mean, and SUV peak. Additionally, SUV metrics such as SUV max and SUV mean have been reported as clinically useful in assessing the activity of osseous lesions and monitoring the treatment response, especially in patients with prostate or breast cancer bone metastases [3].

Vertebral selection and SUV measurement

For each patient, three morphologically normal vertebrae-C4, T6, and L4-were selected to represent cervical, thoracic, and lumbar regions under consistent acquisition conditions, ensuring both anatomical coverage and technical reproducibility for SUV normalization. Vertebrae affected by pathological findings, such as bone metastases, compression fractures, or artifacts (e.g., surgical implants), were excluded. The highest SUV for each vertebra was recorded.

Statistical analysis

EZR software (Saitama Medical Center, Jichi Medical University, Saitama, Japan) was utilized for statistical analysis. The distribution of collected SUV values was assessed using the Shapiro-Wilk test to determine normality. Assuming a normal distribution, the upper limit of physiological uptake was defined as the mean plus 1.64 standard deviations, which corresponded to the 95th percentile in a one-tailed distribution.

Results

Out of a total of 102 vertebrae from 34 patients, 33 were excluded due to abnormal findings. The remaining 69 vertebrae were considered morphologically and functionally normal and included in the analysis. The mean SUV values for each vertebra are shown in Table 1.

**Table 1:** Mean SUV for each vertebra SUV, standardized uptake value

	The mean SUV
C4	7.50 ± 2.13
T6	7.85 ± 1.60
L4	7.47 ± 1.83
All	7.63 ± 1.84

**Figure 2:**
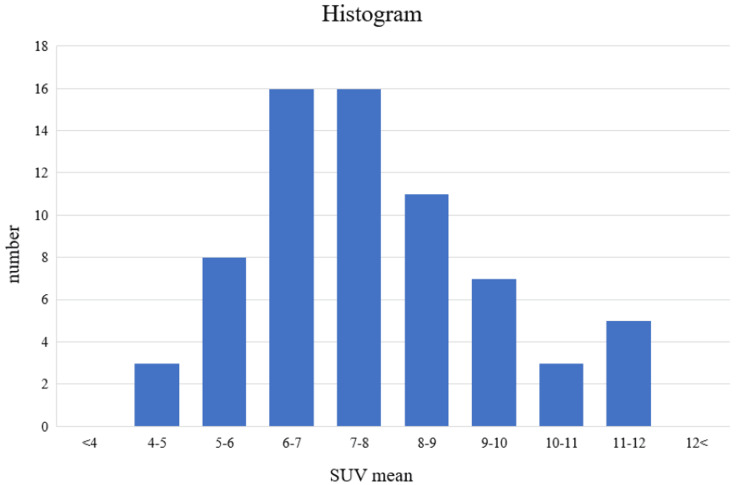
SUV mean histogram This is the SUV histogram for the entire spine. The mean SUV value was 7.63 ± 1.84. SUV, standardized uptake value

From these, the mean SUV across these vertebrae was 7.63 ± 1.84 (Figure 2).

**Figure 3:**
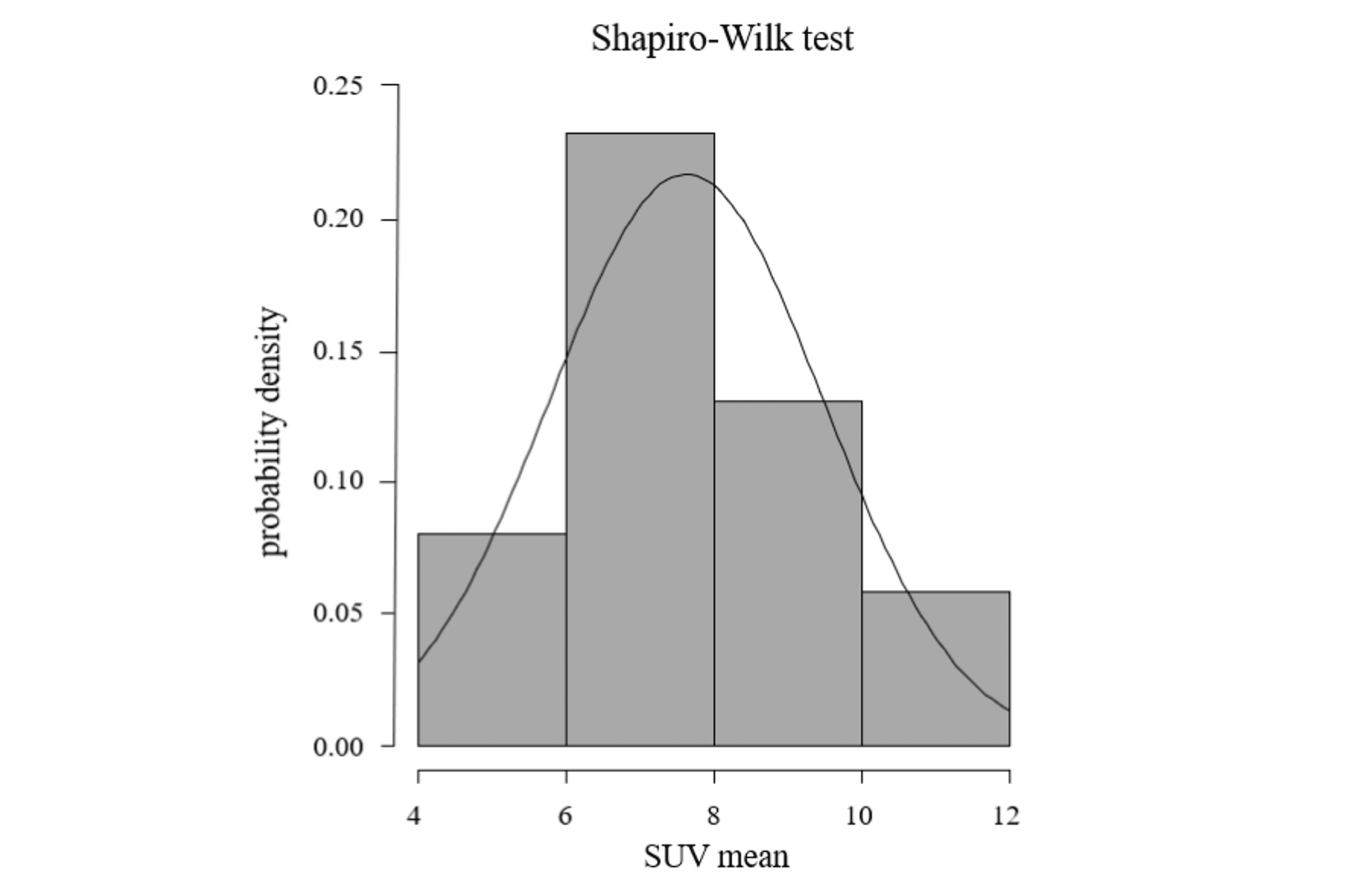
Shapiro-Wilk test The Shapiro-Wilk test yielded a p-value of 0.13, indicating results consistent with a normal distribution.

The Shapiro-Wilk test indicated that the SUV distribution did not significantly deviate from normality (p = 0.13) (Figure 3).

Based on the mean and standard deviation, the SUV threshold was calculated as: mean + 1.64 × SD = 7.63 + 1.64 × 1.84 = 10.65. Beyond establishing this numeric cutoff, the method we applied demonstrates a reproducible approach to deriving thresholds tailored to actual imaging conditions.

This threshold represents the upper boundary of SUV expected in physiologically normal vertebrae processed under the institution's conditions.

## Discussion

This technical evaluation identified an SUV threshold of 10.65 for xSPECT-based GI-BONE analysis, which is higher than the 7.0 threshold reported with conventional SPECT [4]. The increase likely reflects the improved resolution and quantification provided by xSPECT, which enhances tracer uptake values in both normal and abnormal bone.

On clinical evaluation, xSPECT/CT identified more abnormalities and promoted greater diagnostic confidence than standard SPECT, being consistent with the higher SUVs [5]. Also, quantitative SPECT/CT SUVs for metastatic lesions were markedly higher than those for degenerative foci [1]. In our data, the mean “normal” vertebral SUV already approached the old threshold, so a higher cutoff was necessary to maintain specificity. The practical implication is that using a fixed threshold of 7.0 (derived from older techniques) for xSPECT-processed images would likely yield many false positives: labeling normal bone as pathologic. In contrast, by establishing a threshold using patient images that have been processed in accordance with the conditions of our institution, we can improve the clinical relevance and diagnostic accuracy of GI-BONE-based assessments. This approach also supports personalized imaging interpretation, which is essential in the context of diverse imaging platforms and protocols.

A higher threshold may reduce sensitivity for low-uptake sites such as ribs [6]; thus, adaptive or region-specific SUV correction factors should be evaluated in future validation studies to prevent underdiagnosis. Beyond institutional optimization, establishing a robust SUV threshold contributes to the broader standardization of quantitative SPECT. Standardized quantitative parameters are fundamental for multi-center comparison, longitudinal monitoring, and integration into theranostics or response-assessment frameworks. By defining a reproducible reference for normal bone uptake, this study supports the transition of SPECT/CT from qualitative interpretation to fully quantitative imaging, bridging the gap toward PET-level quantification and clinical applicability.

Another important consideration is the potential variability of SUV measurements related to scanner performance and acquisition or reconstruction parameters. Although all scans in this study were acquired on the same xSPECT/CT system under uniform reconstruction settings, SUV values can vary between institutions due to differences in calibration, acquisition time, and reconstruction algorithms. Future multi-center investigations with harmonized imaging protocols and cross-calibration among scanners will therefore be essential to confirm the reproducibility and generalizability of our threshold.

Moreover, the present study had limitations. The sample size of 34 patients may limit the generalizability of the SUV threshold. Additionally, the focus on urological malignancies may mean that the data do not fully represent other cancer types with bone metastases. Thus, future studies with larger, multi-institutional cohorts are needed to validate the findings.

## Conclusions

This study quantitatively defined an institutional SUV threshold of 10.65 for GI-BONE analysis based on xSPECT bone scans, reflecting the higher contrast and improved quantification of xSPECT compared with conventional SPECT. This higher cutoff reflects the improved spatial resolution and quantitative accuracy of xSPECT reconstruction and is tailored to our specific imaging conditions. By defining an empirically derived SUV threshold based on institution-specific SPECT/CT acquisition and reconstruction parameters, our method enhances the diagnostic specificity of GI-BONE quantitation and supports the broader standardization of quantitative SPECT in clinical practice.

As the next step, we will apply this GI-BONE SUV threshold in routine bone scan analyses for urologic cancer patients. The method we used to empirically derive a site-specific threshold can serve as a framework for other institutions to establish their own quantitative reference, thereby contributing to the standardization and broader clinical implementation of quantitative SPECT/CT. Future multi-center validation studies are warranted to confirm generalizability across diverse scanners and protocols.
